
JOURNAL INFORMATION
==============================
NLM Title Abbreviation: Hum Genomics
Journal ID: Hum. Genomics
NLM Journal ID: 101202210

Human Genomics

ISSN: 1473-9542
EISSN: 1479-7364
Publisher: BMC

ARTICLE INFORMATION
==============================
PMCID: PMC5850937
DOI: 10.1186/s40246-018-0138-6
Article ID: 138
Article version: 1
Subjects: Meeting Abstracts

Abstracts from the Human Genome Meeting 2018

Yokohama, Japan, 12-15 March 2018

Electronic publication date: 2018 Mar 9
Publication date: 2018
Volume: 12
Issue: Suppl 1
Issue sponsor: Publication costs for this supplement were not supported by sponsorship.
Electronic Location ID: 9
Copyright: © The Author(s). 2018
License: Open AccessThis article is distributed under the terms of the Creative Commons Attribution 4.0 International License (http://creativecommons.org/licenses/by/4.0/), which permits unrestricted use, distribution, and reproduction in any medium, provided you give appropriate credit to the original author(s) and the source, provide a link to the Creative Commons license, and indicate if changes were made. The Creative Commons Public Domain Dedication waiver (http://creativecommons.org/publicdomain/zero/1.0/) applies to the data made available in this article, unless otherwise stated.
License URL: https://creativecommons.org/licenses/by/4.0/

Conference name: Human Genome Meeting 2018
Conference Acronym: HUGO 2018
Conference location: Yokohama, Japan
Conference date: 12-15 March 2018
issue-copyright-statement: © The Author(s) 2018

==============================
Publisher’s Note

Springer Nature remains neutral with regard to jurisdictional claims in published maps and institutional affiliations.

Download to read this full article text

